# Critical variables regulating age-related anabolic responses to protein nutrition in skeletal muscle

**DOI:** 10.3389/fnut.2024.1419229

**Published:** 2024-08-06

**Authors:** Colleen S. Deane, Jake Cox, Philip J. Atherton

**Affiliations:** ^1^Human Development & Health, Faculty of Medicine, Southampton General Hospital, University of Southampton, Southampton, United Kingdom; ^2^Centre of Metabolism, Ageing & Physiology, MRC/Versus Arthritis Centre of Excellence for Musculoskeletal Research, NIHR Biomedical Research Centre (BRC), University of Nottingham, Royal Derby Hospital Medical School, Derby, United Kingdom; ^3^Faculty of Sport and Health Science, Ritsumeikan Advanced Research Academy (RARA), Ritsumeikan University, Kyoto, Japan

**Keywords:** protein, nutrition, muscle, ageing, exercise

## Abstract

Protein nutrition is critical for the maintenance of skeletal muscle mass across the lifecourse and for the growth of muscle in response to resistance exercise – both acting via the stimulation of protein synthesis. The transient anabolic response to protein feeding may vary in magnitude and duration, depending on, e.g., timing, dose, amino acid composition and delivery mode, which are in turn influenced by physical activity and age. This review aims to: (i) summarise the fundamental metabolic responses of muscle to protein feeding, (ii) discuss key variables regulating muscle anabolic responses to protein feeding, and (iii) explore how these variables can be optimised for muscle anabolism in response to physical activity and ageing.

## Muscle mass regulation in health and ageing

Skeletal muscle plays an integral role in maintaining health throughout the life-course; an illustration being the close links between low muscle mass/strength and all-cause morbidity and mortality. Reduced muscle mass and strength are also predictive of declines in activities of daily living and increased dependence (1). Such functional limitations and dependence have been highlighted as key factors in reducing quality of life in older individuals (2), emphasising the importance of preserving muscle mass to maintain quality of life.

Beyond physical function, muscle is critical to whole-body metabolism through its role as an amino acid (AA) reservoir, the utilisation of fat and glucose, and the storage of glucose as glycogen (3, 4). Skeletal muscle contributes to energy expenditure through various means, including basal metabolism, physical activity and thermogenesis (5, 6). Reduced energy expenditure is associated with increased risk of obesity, metabolic syndrome, type II diabetes and cardiovascular disease (7), meaning it is critical to maintain metabolically active skeletal muscle tissue to sustain energy expenditure. The quantity of skeletal muscle mass relative to body weight has also been shown to be inversely associated with insulin resistance and pre-diabetes even in populations with healthy quantities of muscle mass (8). However, these detrimental effects are most concerning in more vulnerable individuals such as those with age-related declines in skeletal muscle mass and function (sarcopenia) (9), where increased insulin resistance and a lower contribution of muscle mass to total energy expenditure have been associated with increased risk of metabolic disease (10) and type II diabetes (11).

Given the crucial role of muscle in both physical function and metabolic health, maintaining muscle mass and strength throughout the life course is vitally important. Muscle mass exists in a state of constant turnover that is determined by the processes of muscle protein synthesis (MPS) and muscle protein breakdown (MPB) (12). Changes in net balance are primarily driven by changes in MPS, which are approximately four to five times greater than changes in MPB in response to protein nutrition and resistance exercise (RE) (13). This, combined with the greater technical challenge of quantifying MPB compared to MPS, means that research assessing the anabolic effect of nutritional and RE interventions is focused largely on alterations to MPS (14). In young, healthy individuals, MPS and MPB exist in a dynamic equilibrium. Under these conditions, AAs leave the muscle in the postabsorptive state and are utilised, e.g., for hepatic gluconeogenesis or synthesis of proteins in other tissues (4). This is balanced by the synthesis of new muscle protein in the postprandial state when there is excess availability of building block AAs from protein intake (12).

It follows that disruption of this equilibrium in muscle wasting conditions (such as ageing), skews it towards reduced net protein balance, driven largely by reductions in MPS (15). This age-related blunted anabolic response to key stimuli, namely protein feeding (16) and physical activity (17), has been termed “anabolic resistance” (18, 19), which incipiently chips away at muscle mass, contributing to the onset and progression of sarcopenia. While the mechanisms of anabolic resistance remain at large, age-related inflammation (20) and increased splanchnic uptake of AAs (21–23) are purported contributors implicated in reducing the MPS response to protein and activity. Due to age-related anabolic resistance, there is consensus in the literature that the current recommended daily protein intake of 0.75 g protein/kg/day in the UK (24) or 0.8 g protein/kg/day internationally (25) is insufficient for older adults (26–30), which is unsurprising given the recommended protein intake is meant as a guideline for *all* adults regardless of age. As such, recent think tanks and consortia have recommended a protein intake of approximately 1.0–1.5 g/kg/day for older individuals (26, 29), which, in the face of age-associated reductions in appetite (31), may still be achievable without excessive feeding via increasing the proportion of protein in the diet. Similarly, during acute or chronic illness or injury, MPS rates are suppressed and MPB rates may be elevated, resulting in more rapid skeletal muscle atrophy (32). This is concerning in older populations, as the onset and development of sarcopenia may be accelerated due to illness through various disease-mediated mechanisms (33), while prolonged hospital stays and inactivity following injury result in poorer functional outcomes in the long-term (34, 35).

Maximising anabolic potential is necessary to delay the onset and progression of sarcopenia and maintain function and quality-of-life throughout all stages of the life course. In addition to physical activity, the foundation of achieving this is good nutritional practice, namely in relation to dietary protein intake. Numerous variables impact the anabolic effectiveness of protein intake, including, e.g., the timing, type and quality of the protein source delivered (36), alongside external factors such as the combination of feeding with physical activity (12) and the effects of ageing (37). Given this complexity, there is no universal recommendation that can optimise protein intake for all individuals in all conditions. Instead, these variables should be considered carefully and protein feeding adapted to meet the needs of different individuals (30). As such, the aims of this review are to (i) summarise the fundamental metabolic responses of muscle to protein feeding, (ii) discuss key variables regulating muscle anabolic responses to protein nutrition, and (iii) explore how these variables can be optimised for muscle anabolism in response to physical activity and ageing; all in the context of the human literature.

## Temporal anabolic response to, and timing of, protein nutrition

The anabolic effects of protein feeding are temporally regulated. There is approximately a 30–45 min delay between the consumption of a protein source and subsequent increases in MPS; the magnitude of which is approximately 200–300% compared to postabsorptive (i.e., fasted) rates (38, 39). This delay can be attributed to the time taken for digestion of the protein source and subsequent absorption of AAs into the blood before being transported to the target muscle tissue where it acts as both the stimulus and substrate for increases in MPS. When AAs are provided intravenously (i.e., negating the need for digestion), there is still some latency in the MPS response as the AAs are transported to, and accumulate within, the muscle (40). After this period, MPS rates remain elevated for approximately 90 min, beyond which time there is a rapid decline back to postabsorptive rates, a phenomena termed “muscle full” (38). The muscle full effect is consistent with the understanding that muscle hypertrophy in adulthood cannot be achieved in the absence of accompanying physical activity (12) no matter the protein quantity consumed. Instead, replenishment of muscle protein lost during breakdown in the postabsorptive state is the homeostatic endeavor. To date, the mechanisms regulating the muscle full effect remain elusive. The reduction of MPS rates back to baseline occurs despite continued elevated plasma and intramuscular essential amino acids (EAAs), meaning that these are not responsible for MPS resetting. This also cannot be attributed to de-phosphorylation of key mTOR substrate signalling proteins including p70S6K1, 4EBP1 and EIF4G as these have all been shown to remain elevated after MPS returns to baseline (38). One speculated mechanism is endoplasmic reticulum (ER) stress caused by misfolding of proteins (41), which leads to the unfolded protein response (UPR) to limit further translation of misfolded proteins (42). UPR activity has been implicated in skeletal muscle loss (42) and ageing (43), but has also been found to be sensitive to physical activity (42), which could be relevant given the capacity of activity to delay the muscle full effect.

The duration of the refractory period (i.e., the duration before another MPS stimulation may be achieved) is speculated to be ~3–4 h (44); this being based on findings from Witard et al. (45) showing maximal increases in MPS following 20 g protein feeding approximately ~4 h after consumption of a high protein (0.54 g/kg) breakfast. This is also supported by a study in young people during recovery from physical activity, which showed that 20 g of protein feeding every 3 h produced greater increases in MPS over a 12 h recovery period compared to 10 g every 1.5 h and 40 g every 6 h (46). While these results do represent an interesting starting point for investigating the time-course of regaining sensitivity to protein feeding, physical activity has been well documented to increase the magnitude and duration of the MPS response to EAAs – essentially delaying the muscle full effect (47, 48). Consequently, it is possible that the optimal strategy of protein feeding approximately every 3–4 h exhibited in the studies by Witard et al. (45) and Areta et al. (46) may not apply to the rested state due to these alterations in the muscle full phenomenon. Further, it would be useful to consider the potential implications of factors such as ageing on this refractory period in the rested state, as ageing is pertinent to the onset of sarcopenia, meaning that establishing optimal nutritional strategies for the ageing population is highly important. In sum, the anabolic response to protein nutrition is regulated by the muscle full effect, meaning that, to maximise our anabolic potential, it is important to appropriately time protein feeding around the refractory window.

## Protein dose

The optimal protein quantity needed to elicit a maximal anabolic response has been well researched, with a consensus now established (37, 49). In healthy, recreationally active young adults, a per meal dose of roughly 20 g of “high-quality” protein (or 0.24 g/kg), or 10 g EAAs (roughly equivalent to 20 g intact protein) is sufficient to elicit a maximal and transient MPS response (49). Demonstrating this dose–response relationship between protein intake and MPS, 20 g of whey protein elicited greater MPS responses compared to 10 g whey, with no further anabolic effect observed with 40 g whey except increased AA oxidation and ureagenesis (45). Importantly, these protein quantities determined using isolated protein sources such as whey/EAAs, translate into realistic meal-like settings whereby a moderate portion of lean beef, providing ~30 g of protein (~10 g EAAs), elicits maximal MPS, with no further anabolic benefit seen with much larger portions providing ~90 g protein (~30 g EAAs) (50). It should be noted that physical activity may influence the protein dose needed to elicit a maximal anabolic response and is discussed in the “Physical activity” section.

Due to well-established anabolic resistance to protein intake seen with ageing, the protein dose needed to evoke maximal MPS responses is different in older age. A comprehensive retrospective analysis of multiple studies estimated that the dose of protein required to maximally stimulate MPS in older adults is ~68% greater compared to younger counterparts, resulting in a recommendation of 0.40 g protein/kg body mass for older individuals (49). In practice, ∼40 g protein or ∼20 g EAAs would be required to achieve an MPS response in older adults that resembles that of younger adults (49). While the mechanisms regulating anabolic resistance to nutrition remain to be precisely defined, older adults have been shown to exhibit hyperphosphorylation of mTORC1, potentially manifesting as a reduced ability of aged muscle to phosphorylate mTOR and activate MPS in response to protein, and is thus one plausible regulator (51). In the context of lower than maximal doses, it has been shown that whey protein, delivering 14.86 g total AAs, elicits greater muscle protein accrual compared to EAAs, delivering ~6.72 g total (E)AAs, demonstrating the importance of protein *amount* in older adults, and that when given in these doses, the mechanisms of protein accrual may go beyond EAAs (52). In addition to protein dose, recent evidence has highlighted the importance of protein per meal for ageing muscle, by finding that the number of meals with either ≥20 g or ≥ 30 g of protein were significantly associated with greater *m. vastus lateralis* cross sectional area and appendicular lean mass (53). Protein dose across the day must therefore be carefully considered to maximise diurnal muscle anabolism. Thus, in healthy younger adults ~20 g of high-quality protein (e.g., whey) or 10 g EAAs is sufficient to elicit maximal MPS responses, with this amount increasing to ~40 g of high-quality protein or 20 g EAAs to evoke similar anabolic responses in older adults.

## Protein “quality”

According to the World Health Organization, protein quality can be defined by the amount and proportion of individual EAAs that can be absorbed from the diet and used by the body (54). Until recently, protein quality was estimated using the Protein Digestibility-Corrected Amino Acid Score (PDCAAS), which estimates protein quality based on fecal nitrogen, up to a value of 1.0 (55). Due to limitations with the method, PDCAAS has since been replaced by the Digestible Indispensable Amino Acid Score (DIAAS), which scores protein quality based on ileal digestibility and a theoretical reference protein, which results in foods having a similar score to PDCAAS but without truncating the value at 1.0 (55, 56). As such, despite whether evaluated using PDCAAS or DIAAS, protein foodstuff that provides all 9 EAAs such as meat, chicken, fish and dairy (e.g., milk) are all deemed “high-quality” protein sources (55). In depth discussion and comparisons on PDCAAS and DIAAS is beyond the scope of this review, and so we direct the readers to the following rich resources for further reading (36, 55–59).

With respect to muscle, early studies confirming EAAs as the principal nutritional stimulators of MPS (60) were later refined to reveal phenylalanine, valine and leucine as the most anabolically potent of these EAAs (61, 62). Since then, multiple human studies have repeatedly shown leucine as the most potent EAA for stimulating MPS (63, 64), while also demonstrating leucine as the EAA that elicits the most robust anabolic signaling responses mediated via Sestrin2 sensing and the mTORc1–p70S6K1 pathway (65, 66). Combined, these findings have led to the consensus that leucine is a multifunctional EAA, which can act as the main trigger for the initiation of MPS, in addition to being a substrate for the synthesis of *de novo* proteins (64, 65). The importance of leucine in anabolic responses to feeding has been demonstrated both in isolation and in combination with EAAs. In isolation, as little as 3.42 g of leucine has been shown to *maximally* stimulate MPS by ~110% (64). To place this in the context of more traditional protein feeding regimes, a large protein meal of 48 g whey resulted a ~ 150% increase in MPS (38). Comparing these anabolic responses directly over a 2.5 h measurement period (i.e., time taken for peak MPS and return to baseline) results in a similar overall protein accretion, via increases in MPS (64), demonstrating that leucine alone can evoke maximal MPS, at least until other EAAs become rate limiting (63).

As a branched chain amino acid leucine is metabolised within skeletal muscle, implicating its metabolites in muscle anabolism, with one metabolite, β-hydroxy-β-methylbutyrate (HMB), demonstrating anabolic facets. In humans, 3.42 g of free-acid HMB, providing 2.42 g of pure HMB, becomes rapidly bioavailable in plasma and muscle, and has been shown to stimulate MPS (+70%) and inhibit MPB (−57%); the latter in an insulin-*independent* manner (64). While isolated leucine and its metabolite, HMB, are anabolically effective, if provided in isolation repeatedly overtime other EAAs must become rate limiting for MPS (63). As such, leucine enriched amino acid (LEAAs) strategies have been trialed to exploit the anabolic potency of leucine without compromising MPS in the longer term. When compared with a standard feed of 20 g whey, a much smaller LEAAs feed (3 g, 40% leucine) resulted in a similar temporal MPS response, despite greater insulinemia and aminoacidemia in response to whey. This suggests that whey offers no trophic advantage over LEAAs – or in other words – LEAAs are equally anabolic to larger whey doses (67). This was further confirmed using even smaller doses, with only 1.5 g of LEAAs containing 0.6 g leucine, robustly, and possibly maximally, stimulating MPS, with negligible anabolic advantage of greater doses of LEAA (6 g, 40% leucine) or whey (40 g) (68). Similarly demonstrating the anabolic significance of leucine, a lower-protein but leucine-matched feed (10 g) induced similar increases in MPS compared with a higher-protein feed (25 g) (69), indicating that leucine – and not total protein content – is the primary determinant of anabolic responses in muscle. This has important ramifications for certain cohorts who have, for example, reduced appetite (i.e., ageing), whereby leucine-enriched supplements/feeds may represent an advantageous approach to evoke maximal muscle anabolic responses (69). In a further study trialing the efficacy of leucine “top-ups” in humans, 15 g of EAAs compared with 15 g EAAs plus a 3 g leucine top up 90 min after feeding elicited a similar temporal MPS profile, whereby MPS increased until the onset of the “muscle-full” state ~180–240 min after feeding (70). Thus, while leucine can be used effectively to supplement meals containing suboptimal protein levels when leucine is given shortly after adequate EAAs feeds it has no further anabolic effect. The time frame in which protein/EAAs/leucine re-feeding is capable of re-stimulating MPS remains to be defined. Together, these data suggest that the composition of protein/EAAs, notably the presence of leucine, rather than amount of protein/EAAs is most crucial for stimulating muscle anabolism.

## Protein delivery profile

Bioavailability of EAAs in the circulation and subsequently at the muscle tissue is of paramount importance for stimulating MPS and are variables that can be impacted by the protein delivery profile. Skewed protein feeding, where most of the daily protein intake is fed in a single meal, has been proposed as an alternative to the traditional even diet where protein intake is distributed similarly across multiple meals throughout the day. This maybe particularly relevant in older populations who have greater first pass splanchnic sequestration of AAs, resulting in a reduced hyperaminoacidemia in response to protein feeding compared to young individuals (21–23). It should be noted, however, that others have shown similar AA delivery to muscle across age despite a greater first-pass splanchnic sequestration in older age (23). Skewed protein feeding has also been applied to older hospitalised patients over a six-week period and it was suggested that this diet produced greater plasma AA availability (i.e., aminoacidemia) compared to even feeding (71). While this is noteworthy, plasma AA concentrations were only recorded for 3 h following the midday meal, where the even feed diet provided 30% of total daily protein intake, compared to 78% for the skewed diet. Therefore, there is no consideration in these results for any potential reduced hyperaminoacidemia observed following the three other meals where the even protein feed supplied more protein than the skewed protein feed. Other studies have also reported benefits of a skewed protein feed pattern compared to even feeds, with enhanced retention of fat free mass, greater whole body protein turnover and improved nitrogen balance (72). However, this is in contrast to the findings of Mamerow et al. (73), who reported greater 24 h MPS responses with an even protein feed than a skewed protein feed. Importantly, the study by Arnal et al. (72) was done in older individuals (average age 68 ± 1 y), whereas the study by Mamerow et al. (73) was carried out in younger individuals (average age 36.9 ± 3.1 years). The differences in findings may be reflective of the increased first pass splanchnic sequestration of AAs in older people, though this could best be confirmed by a study design with four experimental groups assessing both even and skewed protein feeds in both young and older participants.

Using more direct (i.e., stable isotopic tracers) methods to capture the transient responses to different protein delivery methods, Mitchell et al. (39) reported that young adults consuming EAAs as a single bolus (15 g) displayed rapid aminoacidemia and insulinemia, whereas smaller repeated “pulse” doses (4 × 3.75 g every 45 min) achieved gradual low-amplitude aminoacidemia and blunted insulin responses. Despite the different systemic profiles, the muscle anabolic response was the same across both delivery methods, demonstrated by the identical MPS temporality (i.e., latency period and return to baseline) and similar MPS rates (39). This data suggests that EAA delivery profile is not an important determinant of muscle anabolism and also implies that rapid aminoacidemia is not a key factor for maximising MPS (39). A follow-on study in older adults consuming EAAs as a single bolus (15 g) or as smaller repeated “pulse” doses (4 × 3.75 g every 45 min) reported that bolus feeding resulted in rapid essential aminoacidemia and insulinemia, which was accompanied by robust mTOR signaling (74). By comparison, pulse feeding resulted in a gradual low-amplitude aminoacidemia and diminished insulin responses, with undetectable mTORC1 signaling changes (74). Despite these attenuations, similar MPS responses were observed, where in fact MPS was sustained beyond 3 h following the pulse feed, by which point MPS had returned to baseline in response to bolus feeding (74). As such, in line with the prior study in young adults (39), there was no anabolic benefit of rapid aminoacidemia in older adults, which is despite greater overall EAA exposure and enhanced anabolic signaling. Instead, the benefit of low-grade-sustained EAA exposure elicited by pulse feeding seems to be the apparent delay in the onset of “muscle-full” permitting equal MPS responses, compared to bolus feeding. As such, the data so far suggests that the protein feed delivery method is not a crucial consideration so long as the protein quantity is sufficient to maximise MPS.

## Protein blends

Protein blends are a mixture of two or more protein sources fed simultaneously. These may be different animal proteins (e.g., whey, casein), plant proteins, (e.g., soy, wheat), collagen proteins (e.g., gelatin) or combinations of these (36). Animal protein sources have complete EAA profiles (i.e., contain all 9 EAAs) compared to most plant protein sources, meaning that they produce more robust EAA hyperaminoacidemia and MPS responses, while plant proteins are either (i) incomplete protein sources meaning they contain some but not all 9 EAAs, or (ii) contain all EAAs but in insufficient quantities (e.g., pea protein contains all 9 EAAs but is insufficiently low in methionine and cysteine), but represent (on average) a more sustainable source of protein (36). Collagen proteins, on the other hand, are a rich source of non-EAAs but a poor source of EAAs, thus limiting anabolic potential. For a comprehensive review on protein sources, readers are directed to the following resource (36). Surprisingly few studies have assessed the anabolic role of protein blends in the rested state. This is an important consideration as many individuals, particularly those who are most at risk of sarcopenia such as older adults, are less (or unable to be) physically active (75). Some recent studies have taken this approach when assessing the efficacy of plant-based protein blends compared to milk protein in the rested state (76, 77). Interestingly, these studies have reported that there was no difference in MPS responses between plant-based protein blends and milk protein, despite the milk protein producing a significantly greater increase in plasma EAAs in both studies. Considering the potency of even small doses of leucine, it is perhaps unsurprising that plant-based protein blends containing greater than 1.8 g leucine were able to stimulate a maximal increase in MPS in young individuals. Importantly, the reduced availability of EAAs in plant-based proteins means that large quantities of protein will be required to achieve the same hyperaminoacidemia as can be achieved with animal proteins, potentially creating challenges for older individuals with reduced appetite who may not want to consume more protein, as well as somewhat counteracting the sustainability advantages of these sources. In the context of ageing, protein source is also important to maximising MPS responses with previous findings favouring whey protein over other protein sources such as casein and soy for older adults (78–80). The mechanisms behind the enhanced MPS response are likely two-fold: a combination of the rapid digestion of whey protein and its higher overall leucine content compared to other protein sources. This rapid digestion also elicits more pronounced hyperaminoacidemia, particularly in older individuals who experience greater splanchnic sequestration of AAs.

The interaction between protein blends and acute RE have also been assessed, e.g., where milk (a casein and whey protein blend) elicited a greater MPS response in the 3 h post-exercise period than soy protein (81). Moreover, when extended to a chronic training period over 12 weeks, there was a greater increase in type II muscle fibre area and fat-and bone-free mass in the milk group than the soy group (82). Other studies have demonstrated similar findings in the post-exercise period comparing a 25% soy, 25% whey and 50% casein protein blend with both a protein (83) and a leucine content matched whey protein isolate (84). These studies both demonstrated no differences in MPS rates between the feeds, suggesting that both performed equally. It is worth noting that the study by Borack et al. (83) only found a significant increase from baseline in the whey protein group and not the protein blend group. As the authors suggest, this is likely a result of a higher baseline in the protein blend group caused by high variance rather than reflective of a difference in the capacity of the drinks to stimulate MPS, given the similarity between the performance of both drinks across the postprandial period. Reidy et al. (84) reported a prolonging of the MPS response in the protein blend group reflected by elevated fractional synthesis rate at 2–4 h, which was not observed in the whey protein group. While it is true that, at the 4 h time point, fractional synthesis rates were only higher than baseline in the protein blend group and not the whey protein group, there were no differences in MPS rates between the groups at any given time or when analysed across the entire 4 h postprandial period. Any suggestion of a prolonging effect of a protein blend based on these findings should be cautiously interpreted, but it is noteworthy that this may somewhat corroborate the findings of Hartman et al. (82). Overall, the current evidence suggests some promising applications for protein blends, including those containing plant-proteins, to produce robust MPS responses as individual protein sources and animal protein blends.

## Physical activity

The capacity to go beyond muscle maintenance with nutrition and achieve muscle growth (hypertrophy) is dependent on the addition of contractile activity, particularly RE (85). RE essentially shifts the muscle full set-point to the right when in proximity with protein nutrition (12), increasing both the duration (86) and magnitude (87) of the MPS response. Notably, RE in the postabsorptive, fasted state, increases muscle protein turnover owing to a ~ 100% increase in MPS rates and a ~ 50% increase in MPB rates (88). Thus, net protein balance becomes less negative in the postabsorptive state following RE, but without the provision of protein, RE does not produce a positive state of protein balance. This highlights that neither protein nutrition nor exercise alone are sufficient to achieve hypertrophy; it is the synergistic combination of these anabolic stimuli that is paramount to increasing muscle mass.

Research points to the timing of protein intake in relation to exercise not being the most important factor in hypertrophy or strength gains, with most hypertrophic differences likely explained by the quantity of protein intake (89). This is perhaps because the enhanced anabolic window achieved through RE persists for up to 48–72 h, meaning that sufficient EAA provision throughout this time period will still produce a robust increase in MPS (90). This is not to suggest that there is no effect of the timing of protein intake, and reflecting this, previous work has shown greater increases in strength and hypertrophy following 12 weeks of RE training with post-exercise protein intake compared to 2 h post-exercise protein intake in older individuals (91). Perhaps more important than the timing of the first protein feed relative to a RE bout is to take full advantage of the enhanced anabolic window within this 48–72 h post-exercise period with repeated protein feeds. As already highlighted, there is a refractory period in response to protein feeding following RE in young, trained individuals which is approximately 3 h in duration (86). These findings should be considered in the context of nutritional practices and recommendations, as a large proportion of the population will typically consume three protein-containing meals a day with upwards of 6 h between protein feeds, which is clearly suboptimal. Further, protein consumption is often skewed between meals, with many individuals consuming less protein at breakfast than other meal times, which is shown to result in reduced MPS rates (73), and may be associated with reduced muscle mass and strength. That said, further research is needed (92), particularly in light of contradictory data in older adults demonstrating no significant changes in post-absorptive MPS over 24 h when protein was consumed evenly or skewed throughout the day (93). In the 48–72 h post-exercise window, prolonged postabsorptive periods are essentially wasting some of the anabolic potential from RE training, and feeding following an overnight fast should provide adequate protein to achieve a state of anabolism following prolonged overnight MPB. Therefore, it is critical to regularly consume an adequate amount of protein roughly every 3 hours where possible to maximise hypertrophy following RE.

Regarding dosing, ~20–30 g of high-quality protein is required following RE to maximally stimulate MPS, with excess protein intake beyond this catabolised via AA oxidation (45, 94, 95). There is clearly capacity for reducing this quantity with leucine enriched protein feeds, with 6 g of LEAA supplements able to stimulate similar post-RE MPS rates up to 4 h compared to 40 g whey protein feeding in older women (68). Although these findings were not replicated in the study by Churchward-Venne et al. (63), who found that only 25 g whey protein post-exercise was able to sustain elevated MPS rates over 3–5 h, whereas 6.25 g whey protein supplemented with either leucine or EAAs did not. This disparity between studies is perhaps surprising, as the study by Wilkinson et al. (68) was carried out in older women while the study by Churchward-Venne et al. (63) assessed young male participants. Given the established anabolic resistance that accompanies ageing, it would perhaps be expected that the younger participants would have had a more sustained MPS response even with the lower dose of whey protein fortified with either EAAs or leucine. Potentially, there was no sustained anabolic response to the EAA enriched whey protein feed in this group due to the low leucine content of this feed (0.75 g) despite the high overall EAA content. However, this does not apply to the leucine enriched whey protein feed, which had a comparable leucine content to the feeds in the study by Wilkinson et al. (68). Instead, the difference in post-exercise assessment durations (5 h in Churchward-Venne et al. (63) and 4 h in Wilkinson et al. (68)) may account for some of this disparity, as it is possible that MPS remains elevated under post-exercise conditions with lower quantity leucine fortified feeds, for approximately 4 hours before rapidly declining to baseline. Additionally, it could also be that the differences in lower leg RE protocols between the studies may explain some of the disparity in findings. The study by Wilkinson et al. utilised 6 × 8 repetitions of unilateral leg extensions, compared to 4 × 10–12 repetitions of both unilateral leg extensions and leg press. The addition of the leg press exercise would have resulted in the stimulation of additional muscle groups not utilised in leg press, resulting in a greater post-exercise AA demand compared to leg extension alone, which perhaps could not be sufficiently met by the low dose leucine or EAA feeds. Therefore, lower dose leucine fortified protein may be a robust post-exercise protein source for prolonged anabolism, particularly in older individuals who have reduced appetites and would subsequently prefer smaller protein feeds (31).

While there is general consensus regarding the maximal magnitude and duration of the MPS response to protein feeding following exercise, it was reported that there are greater increases in whole body net protein balance with 70 g mixed meal protein intake compared to 40 g following RE training (96). However, this same study reported no differences at the muscle level, with no effect of meal size, or even exercise, on MPS rates. The lack of effect of exercise can likely be attributed to measuring MPS over a seven-hour post-exercise period, as the peak in MPS rates that would be expected from RE training and protein feeding would largely be masked by prolonged periods of lower MPS rates based on the muscle-full phenomenon. More importantly, post-exercise MPS rates being the same between the 40 g and 70 g protein feed brings into question the relevance of the findings of greater net protein balance with the higher protein feed. Kim et al. (96) suggest that the anti-catabolic benefits of higher protein intakes are important but given that all measures made were of whole-body protein turnover, it is unknown how much of this breakdown can be attributed to muscle compared to other tissues, with the gut being a primary candidate for this. Other studies have reported similar findings following endurance exercise, with a 45 g protein feed producing a significantly greater whole body protein balance than 30 g, but similarly to RE, with no differences in myofibrillar fractional synthesis rates between the protein feeds beyond 30 g (97). Overall, the relevance of a greater whole body protein balance in the absence of any further increases in MPS needs to be considered. It may not be advantageous to compromise other feeds with suboptimal protein quantities that do not produce robust increases in MPS, in order to provide more of a post-exercise pulse feed which may produce greater whole-body protein balance but does not provide further benefits at the muscle level.

Only a single study has suggested that there is no upper limit in magnitude or duration of the anabolic response following RE, reporting a dose-dependent relationship between quantity of protein feeding and increases in myofibrillar MPS rates (98). In this study, it was reported that 100 g protein feeding stimulated greater increases in post-exercise MPS rates than 25 g protein over a 12 h post-exercise period. These findings contrast with previous research showing that post-exercise MPS rates are maximally stimulated with approximately 20 g of protein, with no further increase in MPS with 40 g protein (45, 95). One potential explanation for the differences in these findings could be the type of exercise employed. The study by Trommelen et al. (98) assessed MPS following whole body RE, whereas the studies by Moore et al. (95) and Witard et al. (45) used leg based RE. There is some precedence for this, with previous research indicating that 40 g of protein feeding may be more effective at increasing MPS than 20 g of protein feeding following whole body RE training (99). This is likely caused by the increased demand for AAs following whole body compared to isolated leg-based training, with the 20 g protein dose potentially not supplying enough AAs to meet the demands of the greater number of muscles utilised in whole body RE. It should also be noted that the adults recruited were untrained, which may also contribute to the observed anabolic response, which is known to be attenuated in trained individuals (100). However, these explanations are speculative, and there is currently no study directly comparing MPS responses to different protein doses following different types of RE. Moreover, this study likely simply reflects “on / off / on” of MPS in line with muscle full – due to the lingering large quantities of EAA in the circulation owing to gradual oxidative elimination.

Indeed, the suggestion from Trommelen et al. (98) there is no upper limit in magnitude or duration of the anabolic response to protein feeding should be interpreted with caution. The authors observed a ~ 20% increase in MPS rates in the 0–4 h post-exercise period when comparing 100 g protein feeding to 25 g, which matches the 20% increase also observed in the study by Macnaughton et al. (99) in the 0–5 h post-exercise period when comparing 40 g protein feeding to 20 g, with both studies using whole body RE. This would indicate that there is indeed an upper limit in magnitude of the MPS response, as MPS peaked at approximately the same relative increase compared to a lower dose (40 g vs. 20 g and 100 g vs. 25 g protein, respectively (98, 99)) and absolute value (approximately 0.06%/h), following 100 g protein feeding compared to 40 g protein feeding (98), both following whole body RE. Regarding duration, there was no difference in MPS rates between the 100 g protein group and the 25 g protein group between 8 and 12 h post-exercise, despite the fact that only the 100 g protein group was significantly elevated compared to the 0 g protein group. Given that there was no difference between the two protein feeds over this later time period, the physiological reality of this supposed extended duration of anabolism should be questioned. This is particularly important as the authors suggest that this provides mechanistic insights into the potential benefits of larger, less frequent protein feeding patterns, which is directly in contrast to the findings of Areta et al. (46). There may be some validity in assessing even more doses with higher protein quantities as an extension to the findings of Areta et al. (46) based on the results of Trommelen et al. (98). However, given that four 20 g protein feeds were more efficacious than two 40 g protein feeds (46), it would be surprising to see a shift in favour of an even higher dose, but less frequent protein feed. In sum, RE is a key stimulator of muscle anabolism, but the intricacies of maximising the MPS response are still debated. Repetitive protein feeding containing EAAs/leucine within the 48–72 h post-exercise period should be the primary aim of anyone pursuing muscle hypertrophy, with any additional benefits of large quantities of protein intake (> 40 g) in response to whole body RE requiring further validation.

## Conclusion and future directions

**Figure 1:**
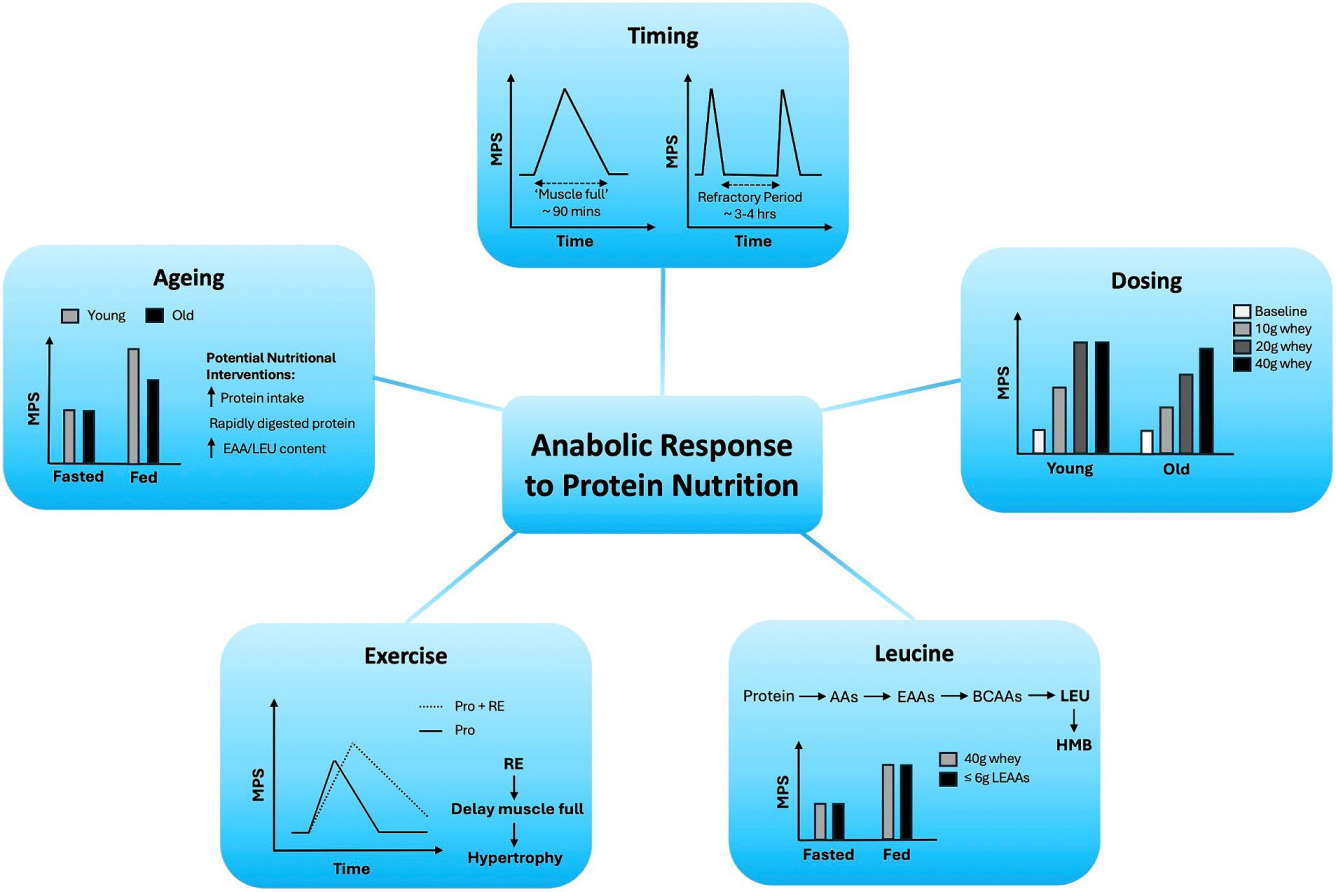
Variables regulating age-related anabolic responses to protein nutrition in skeletal muscle.

Protein nutrition is essential for the maintenance of skeletal muscle mass across the lifecourse and for the growth of muscle in response to RE via the stimulation of MPS. Many critical variables contribute to the duration and magnitude of this MPS response, including the protein dose, timing, EAA/leucine content, and delivery method, which are further impacted by age and exercise, all of which we have summarised in Figure 1.

Based on the reviewed evidence, we provide the following highlights, which contain practical recommendations for, and relevant to, protein nutrition practice:

- Maximal MPS can be achieved with ~20 g high quality protein (e.g., whey) or 10 g EAA in young healthy weightbearing adults.
- Older adults display anabolic resistance to protein nutrition (and exercise), requiring larger amounts of protein, ~40 g high quality protein or 20 g EAA, to elicit a maximal MPS response.
- As an anabolic signal and substrate, small doses of leucine (3 g) can evoke a maximal MPS response. This has significant application in cohorts who cannot, or do not, consume sufficient protein throughout the day to stimulate maximal MPS (e.g., older adults).
- Animal-delivered protein sources contain all EAAs in high quantities, eliciting robust MPS responses. By relative comparison, plant-derived protein sources contain lower EAA levels and in some cases do not contain all EAAs, eliciting less robust MPS responses. Nonetheless, with appropriate protein blending, plant-derived protein feeds can elicit maximal MPS.
- The delivery method of protein feeds (i.e., bolus versus pulse) is not a major determinant of the MPS response, so long as sufficient protein is consumed.
- Following a protein feed, the duration before which another MPS stimulation may be achieved (i.e., the refractory period) is estimated to be ~3–4 h but remains to be precisely defined.
- Consuming a protein feed in close proximity to exercise will ensure a maximal MPS response, although it is not critical as long as sufficient protein is consumed within the 72 h post-exercise period.
- The current protein recommendation of 0.75–0.8 g protein/kg/day is insufficient for older adults and should be increased to 1.0–1.5 g/kg/day.

In addition to these practical tips, we have highlighted future research directions which we consider worthy of research attention in Table 1.

**Table 1:** Future research directions regarding variables regulating age-related muscle anabolic responses to protein nutrition.

Directly investigate the role of the unfolded protein response in regulating the muscle full response
Precisely define the refractory period timeline in healthy young and ageing scenarios
Characterise the anabolic impact of alternate protein sources at rest
Understand whether skewed diurnal protein intake impacts muscle mass/strength
Test the efficacy of chronic bolus versus pulse diets for muscle anabolism in older adults
Re-define the upper limit of magnitude and duration of the MPS response to different protein doses
Characterise the effects of chronic consumption of protein blends, particularly those containing plant proteins, on longer term MPS and muscle mass
Update recommended protein intakes for older adults to reflect that 0.75-0.8 g/kg/day is insufficient for muscle mass maintenance

## Author contributions

CD: Conceptualization, Investigation, Project administration, Writing – original draft, Writing – review & editing. JC: Conceptualization, Investigation, Writing – original draft, Writing – review & editing. PA: Conceptualization, Funding acquisition, Investigation, Project administration, Writing – original draft, Writing – review & editing.
